# 2061 Acellular hyaluronic acid scaffold with growth factor delivery for cartilage repair in a large animal model

**DOI:** 10.1017/cts.2018.43

**Published:** 2018-11-21

**Authors:** Anthony R. Martín, Jay M. Patel, Hannah M. Zlotnick, Mackenzie L. Sennet, James L. Carey, Robert L. Mauck

**Affiliations:** 1 University of Pennsylvania School of Medicine, Philadelphia, PA, USA; 2 University of Pennsylvania, Philadelphia, PA, USA; 3 Translational Musculoskeletal Research Center, Medical Center, University of Pennsylvania, Philadelphia, PA, USA

## Abstract

OBJECTIVES/SPECIFIC AIMS: Focal cartilage injuries of the knee joint are common and present a treatment challenge due to minimal intrinsic repair. Cartilage tissue engineering techniques currently used in clinical practice are expensive, cumbersome, and often ineffective in patients with mechanical or medical comorbidities. To address these issues, we developed an acellular nanofibrous scaffold with encapsulated growth factors designed to enhanced articular cartilage repair. Our goal is to evaluate this technology in vitro and pilot a large animal model for eventual translation into human subjects. METHODS/STUDY POPULATION: Hyaluronic acid (HA, 65 kDa) will be methacrylated (~40% modification, MeHA) and conjugated with cell-adhesive (RGD) groups. A solution of 4% wt/vol MeHA, 2% wt/vol polyethylene oxide (900 kDa), 0.05% wt/vol Irgacure 2959, and 0.005% wt/vol stromal cell-derived factor-1α (SDF-1α) and/or transforming growth factor-β3 (TGF-β3) will be prepared in ddH_2_O. The solution will be electrospun onto a rotating mandrel to achieve a dry scaffold thickness of 0.5 mm. The scaffold matt will be UV cross-linked and 5 mm-diameter samples will be cut out. Four groups of scaffolds will be prepared: MeHA, MeHA+SDF, MeHA+TGF, MeHA+SDF+TGF. All groups will be evaluated for fiber diameter, swell thickness, equilibrium compressive modulus, degradation rate, and growth factor release rate over 4 weeks (n=10). Scaffolds will also be seeded with juvenile porcine MSCs (5×104) in 200 μL of medium incubated for 24 hours. Seeded scaffolds will be evaluated for equilibrium compressive modulus, cell infiltration, and chondrogenesis at 4 and 8 weeks (n=10). Scaffolds will then be evaluated in a juvenile Yucatan minipig cartilage defect model. In total, 6 animals will undergo bilateral knee surgery to create four 4 mm-diameter full-thickness cartilage defects in each trochlear grove. All defects will receive microfracture to release marrow elements. Each knee will receive 2 scaffolds of the same group (replicates) with paired microfracture controls, resulting in a sample size of 3. Animals will be sacrificed at 12 weeks and defects will be evaluated via non-destructive indentation testing for mechanical properties, microCT for defect fill and subchondral bone morphology, and histology for ICRS II Visual Histological Assessment Scoring. RESULTS/ANTICIPATED RESULTS: Our preliminary studies have shown reliable replication of electrospun MeHA scaffolds. We anticipate cross-linking density to correlate positively with compressive modulus, and negatively with swell thickness, degradation rate, and growth factor release rate. We anticipate the addition of SDF-1α and TGF-β3 to increase cell infiltration and chondrogenesis, respectively, within seeded scaffolds. Similarly, we expect minipig defects treated with growth factor-releasing scaffolds to show greater mechanical properties, defect fill, and ICRS II score compared with MeHA scaffolds without growth factor. DISCUSSION/SIGNIFICANCE OF IMPACT: This study has the potential to show how an HA-based cell-free scaffold can be augmented with 2 growth factors that act synergistically to improve cartilage repair in a large animal model. This technology would improve upon the cell-free scaffolds already used clinically for autologous matrix-induced chondrogenesis and is directly translatable.

